# Supplementary material for: Placenta segmentation in ultrasound imaging: Addressing sources of uncertainty and limited field-of-view
Source: Med Image Anal. Author manuscript; Available in PMC 2023 Jan 3. (PMC7614009; doi:10.1016/j.media.2022.102639)
Supplement: Appendices [file EMS157952-supplement-Appendices.pdf]

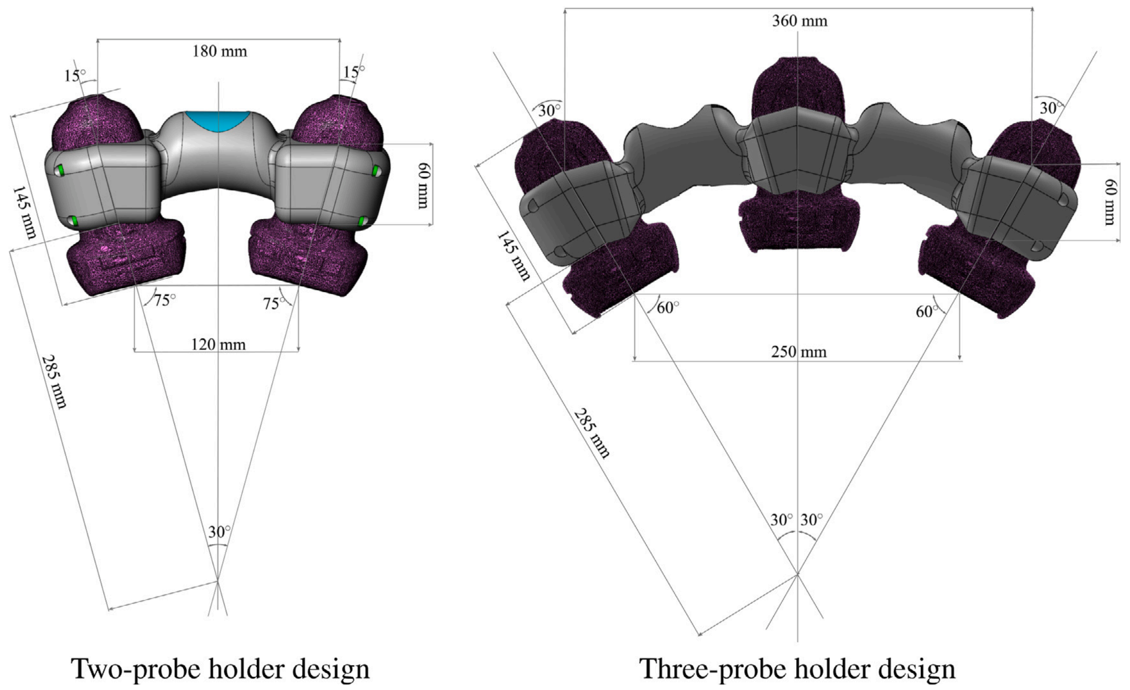

Fig. A.10. Design of a custom-made multi-probe holder for fetal imaging. The probes are fixed in an angle of 30° to each other to ensure a large overlap of the field-of-view. The system is flexible in the sense that it allows the use of two (left) or three (right) probes simultaneously.

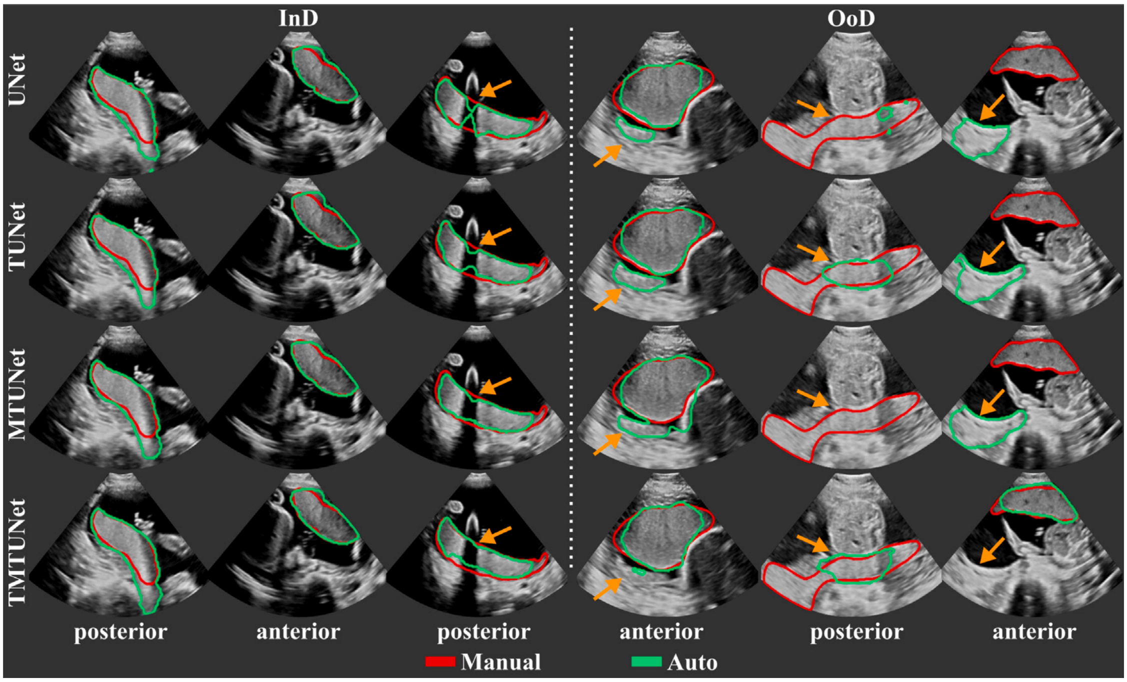

Fig. B.11. Examples of automatic placenta segmentations obtained by models *UNet*, *TUNet*, *MTUNet* and *TMTUNet* for *in-distribution* (InD) and *out-of-distribution* (OoD) test data. The orange arrows indicate areas with segmentation errors and differences between the models. (All images are 3D volumes, central 2D slices are shown.)

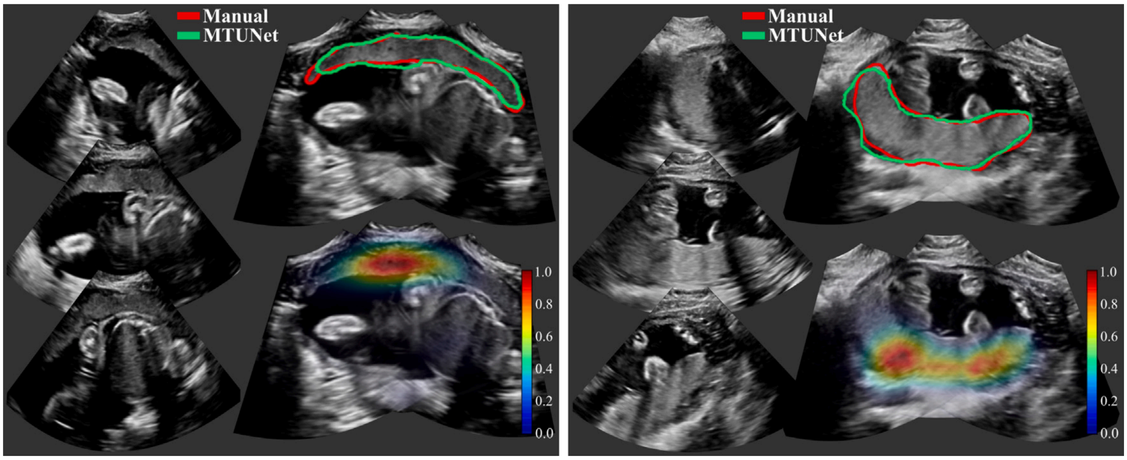

Fig. B.12. Four examples of multi-view images, each showing three individual images (left) and fused images with manual (in red) and automatic segmentation (model *MTUNet* in green) (top right) and combined attention maps (bottom right). (All images are 3D volumes, central 2D slices are shown.)

Table A.5
Data splits for five folds in training, validation and testing sets for the segmentation dataset $\mathcal{I}^S$ and the classification dataset $\mathcal{I}^C$. For $\mathcal{I}^S$, the number of images are given for anterior (ant.) and posterior (post.) placentas. For $\mathcal{I}^C$ additionally the number of images with no placental tissue visible (none) are reported.

	Segmentation data $\mathcal{I}^S$						Classification data $\mathcal{I}^C$								
	Training		Validation		Testing		Training			Validation			Testing		
	ant.	post.	ant.	post.	ant.	post.	ant.	post.	none	ant.	post.	none	ant.	post.	none
Fold 1	90	90	34	24	30	22	286	290	241	101	64	49	89	55	29
Fold 2	90	90	33	19	31	27	276	295	267	111	53	34	89	61	18
Fold 3	90	90	34	21	30	25	285	298	240	102	51	48	89	60	31
Fold 4	90	90	33	19	31	27	288	284	261	99	52	11	89	73	47
Fold 5	90	90	32	23	32	23	287	296	267	98	55	19	91	58	33

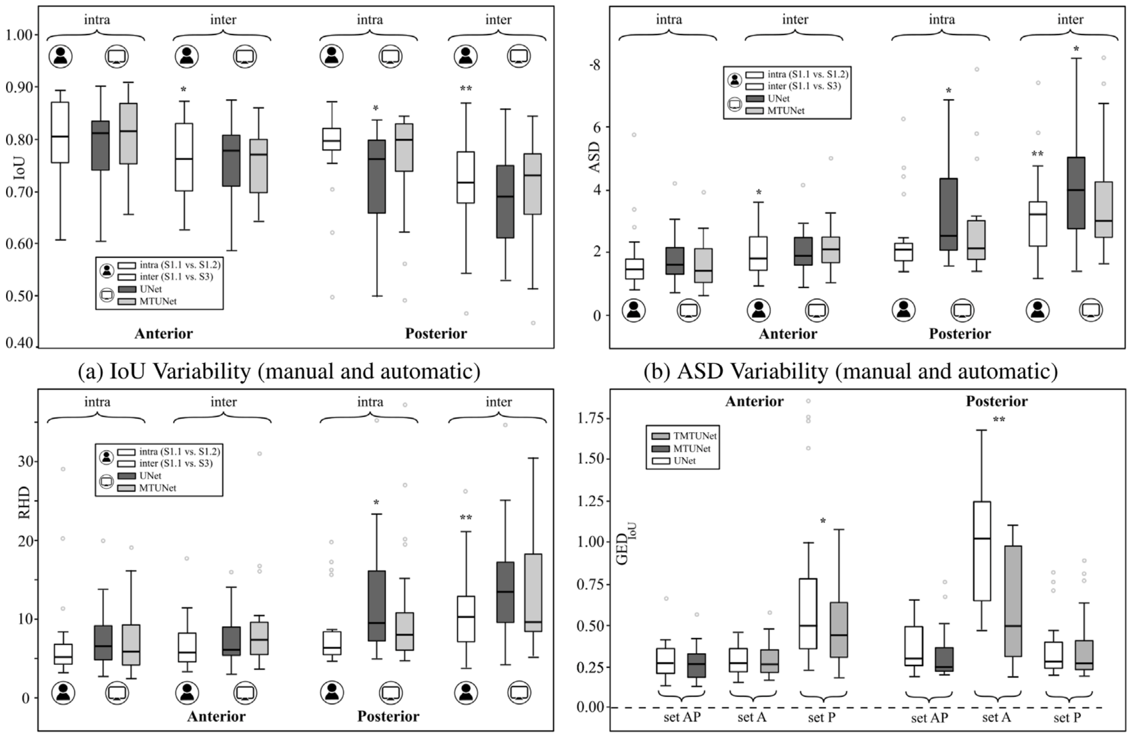

(a) IoU Variability (manual and automatic)

(b) ASD Variability (manual and automatic)

(c) RHD Variability (manual and automatic)

(d) Uncertainty in distributions

Fig. B.13. (a)–(c) Variability among manual and automatic segmentations. The agreement of possible segmentation is measures using (a) the Intersection-over-Union (IoU), (b) the average surface distance (ASD) and (c) the robust Hausdorff distance (RHD). Manual: S1.1 vs. S1.2 (intra) and S1.1 vs. S3 (inter); *UNet/MTUNet*: S1.1 vs. *UNet/MTUNet* (intra) and S3 vs. *UNet/MTUNet* (inter). (b): The difference in distributions between manual annotations from three raters and automatic segmentations from models *UNet*, *MTUNet*, and *TMTUNet* with MC dropout is measured by the Generalized Energy Distance using IoU as distance measure. This is compared for models trained on sets *A, P* and *AP* and tested on both anterior and posterior placentas. Statistical significance between *UNet* and *MTUNet/TMTUNet* is indicated by * (moderate effect size) and ** (strong effect size).

A.1. Probe holder design

Fig. A.10 shows the design of the two- and three-probe holder with measurements in mm. The initial design was developed on a fetal phantom in the second trimester (Kyoto Kagu Space-fan CT), and subsequently optimized with regard to comfort and usability in a clinical setting by scanning pregnant volunteers. The result is a flexible system which allows the use of two, three, or even four probes (not used in this study). We fixed the angulation between the probes so that the FoV can be extended with a known spatial alignment of the images. We chose an angle of 30° which empirically showed to angulate the probes sufficiently to maintain contact between the probe's surface and maternal skin. However, other configurations are possible.

A.2. Data

We perform a 5-fold cross-validation and each fold divides the patients in a test, training and validation set. In each fold, approximately 60% of the data $\mathcal{I}^S$ is used for training, and 20% for both validation and testing. Different folds had different amount of images for validation and testing (up to 10%) because of the heterogeneity of the data: each patient had a different number of images, with and without manual segmentations, and with and without placental tissue. However, we made sure that the images from individual patients were not distributed across training/validation/testing sets, the number of training images with segmentations is always the same for posterior and anterior placentas, and that each patient with manual segmentations is exactly once part of a test set.

Details about the data distribution in the folds can be found in Table A.5.

Appendix B. Results

B.1. Placenta segmentation - Single images

Fig. B.11 visualizes examples comparing the segmentation when the images was InD or OoD data. Multi-task models, especially *TMTUNet* (row 4) show a more robust performance with respect to OoD data. Only *TMTUNet* is able to localize correctly the placenta in these OoD examples. Also, *MTUNet* and *TMTUNet* are more robust to image artifacts, such as shadows, which is shown in InD, last example.

B.2. Placenta segmentation - Multi-view images

Additional exemplary multi-view images are shown in Fig. B.12 with corresponding placenta segmentations with *MTUNet* and combined attention maps. The placenta is better visualized in the multi-view images with reduced image artifacts and an extended FoV. The multi-task model *MTUNet* provides an accurate segmentation and the combined attention maps localize well the placenta.

B.3. Variability and uncertainty

We investigated the inter- and intra-observer variability for the manual annotation of placental tissue in 3D US. In each fold, we use a subset of the test set, for which three manual annotations are available. Fig. B.13(a)–(c) show the agreement of the segmentations as measured by IoU, ASD and RHD, respectively, and Fig. B.13(d) the difference in manual and automatic distributions (as a measure of uncertainty) measured by the Generalized Energy Distance using the Intersection-over-Union (IoU).
